# Adapting the WHO Behavioural and Social Drivers of Vaccination (BeSD) tools and Vaccination Attitudes Examination (VAX) scale for pregnant women in South Africa: Insights from a mixed-methods pilot study

**DOI:** 10.1371/journal.pone.0334854

**Published:** 2025-11-05

**Authors:** Imen Ayouni, Jennifer Nyawira Githaiga, Edina Amponsah-Dacosta, Benjamin M. Kagina, Rudzani Muloiwa

**Affiliations:** 1 Department of Paediatrics and Child Health, Red Cross War Memorial Children’s Hospital, University of Cape Town, Cape Town, South Africa; 2 Vaccines for Africa Initiative and NITAG Support Hub, School of Public Health, Faculty of Health Sciences, University of Cape Town, Cape Town, South Africa; 3 Division of Social and Behavioural Sciences, School of Public Health, Faculty of Health Sciences, University of Cape Town, Cape Town, South Africa; Faculdade Arthur Sa Earp Neto Faculdade de Medicina de Petropolis, BRAZIL

## Abstract

**Background:**

Understanding vaccine attitudes, knowledge, and perceptions is critical for improving vaccine uptake, particularly among vulnerable populations such as pregnant women. This study aimed to test and adapt quantitative survey tools and an interview guide for assessing maternal and childhood vaccine attitudes among pregnant women in the South African context.

**Methods:**

We conducted a mixed-methods pilot study among pregnant women attending antenatal care at two public hospitals in Cape Town, South Africa, between September 2023 and January 2024. Quantitative data were collected using a self-administered questionnaire on tablets, incorporating the adapted BeSD and VAX tools, while qualitative data were gathered through semi-structured interviews. Additionally, the quantitative questionnaire contained the General Vaccine Attitude Survey questions developed by the World Health Organization Strategic Advisory Group of Experts on Immunization (WHO SAGE) Working Group on Vaccine Hesitancy and a validated tool on knowledge of influenza and attitudes toward influenza vaccination during pregnancy. Adaptations to the tools were informed by participant feedback and pretesting.

**Results:**

Eighty pregnant women agreed to participate in the quantitative survey, one participant dropped out of the study and seven completed follow-up qualitative interviews. The tools were well received, with participants reporting ease of use and understanding. Minor linguistic adjustments improved clarity, and participants identified healthcare providers as key influencers in vaccine decision-making. Knowledge gaps regarding maternal vaccination and concerns about vaccine safety during pregnancy were prominent. The mixed-methods approach provided complementary insights into the tools’ applicability and participants’ attitudes.

**Conclusions:**

This pilot study demonstrated that the WHO BeSD tools, the VAX Scale, the General Vaccine Attitude Survey, and the questions on knowledge of influenza and attitudes toward influenza vaccination during pregnancy, with minor adaptations, are feasible and acceptable for use in South Africa. These findings support their application in larger studies to explore maternal vaccine confidence and decision-making. Addressing knowledge gaps and leveraging trusted sources of information is critical to enhancing vaccine uptake in similar settings.

## Introduction

Infectious diseases are associated with high morbidity and mortality among pregnant women, their fetuses, and infants. During pregnancy and in the first few months of life, mothers and babies experience a naturally lowered immune response, making them more susceptible to various infectious diseases [1–3].

In recent years, maternal immunization has gained significant attention from the scientific community. Numerous observational studies have consistently shown that vaccinating women during pregnancy is not only safe but also highly effective as a public health intervention. This strategy provides protection not just for the expectant mother, but also for her developing baby in the early months of life [4–8].

The effectiveness of any vaccination program is related to how well the at-risk population understands the benefits of vaccines and how they work. Insufficient information or misinformation can lead to lower vaccine acceptance among vulnerable groups, such as pregnant women. Understanding their attitudes, experiences, and any concerns or barriers they face is essential, as these factors can significantly influence their decisions regarding vaccination during pregnancy [1,4,9–11]. Additionally, vaccine uptake relies not only on the vaccines’ availability and accessibility; other important factors are significant, particularly mothers’ knowledge and attitudes toward maternal and routine childhood vaccines. Personal factors, such as maternal knowledge and attitudes toward vaccination, can greatly influence whether mothers vaccinate themselves and have their children vaccinated, even when services are available and accessible [12–15]. Understanding how personal beliefs and experiences influence vaccine decision-making among pregnant women is crucial for enhancing vaccine acceptance and coverage in both routine and outbreak contexts. However, most studies have primarily been conducted in high-income countries, resulting in limited data and application of these tools among pregnant women in low- and middle-income countries (LMICs), particularly in African countries [16]. The limited data and research on maternal vaccination in LMICs, especially in African countries, impede the development of context-specific interventions to improve vaccine acceptance. Addressing this gap is crucial for designing effective strategies to increase vaccination coverage among pregnant women and ensure the successful implementation of recommended vaccines in routine care. Therefore, this study will for the first time test the feasibility of the General Attitudes Toward Vaccine Survey questions developed by the WHO SAGE working group on vaccine hesitancy [17]; the Vaccination Attitudes Examination (VAX) Scale [18]; the WHO Behavioural and Social Drivers of Vaccination (BeSD) COVID-19 and Childhood Vaccination survey tools [19]; and a validated survey tool assessing knowledge of influenza and attitudes toward influenza vaccination during pregnancy [20–24] among pregnant women in Cape Town, South Africa. Besides, we aimed to assess if these tools are deemed understandable and acceptable by the study participants.

## Methods

### Study design and setting

A pilot study was conducted to assess the feasibility, acceptability, and usability of the adapted tools in preparation for a prospective mixed-methods cohort study.

We conducted a mixed-method cross-sectional pilot study in the Cape Metro West Region in Cape Town, South Africa to test and adapt the General Attitudes Toward Vaccine Survey questions developed by the WHO SAGE working group on vaccine hesitancy [17]; the Vaccination Attitudes Examination (VAX) Scale [18]; and the WHO Behavioural and Social Drivers of Vaccination (BeSD) COVID-19 and Childhood Vaccination survey tools [19]. In addition, we have included a previously validated questionnaire assessing participants attitudes toward vaccination during pregnancy [20–24]. The cross-sectional sample population was randomly drawn from pregnant women attending their routine antenatal visits at secondary and tertiary public hospitals between September 2023 and January 2024.

The Human Research Ethics Committee (HREC) at the Faculty of Health Sciences, University of Cape Town (HREC REF 011/2023), approved this study. The research team provided participants with an information sheet and informed them that their participation was voluntary. They were also told that they could withdraw from the study at any time and that their decision on whether to participate would not affect the care they received at the clinic. Pregnant women who agreed to participate signed a written informed consent form. Participants who agreed to participate in the semi-structured interviews signed an additional consent form.

### Study population and recruitment

Pregnant women aged 18 years and above presenting to antenatal clinics at these health facilities were invited to participate in the study regardless of the pregnancy trimester. Pregnant women under 18 years, who were not South African citizens or residents of the city of Cape Town, and who did not speak English, IsiXhosa, or Afrikaans were excluded from the study. In addition, pregnant women who were unable to provide consent forms were excluded.

A trained research assistant approached pregnant women while they were waiting for their appointments at the antenatal clinics, screened participants for eligibility, Pregnant women who agreed to participate signed a written informed consent form.

Given the pilot nature of this study, a sample size calculation was not performed; however, we considered 10% (n = 81) of the estimated sample size (n = 810) for the prospective mixed-methods cohort study this pilot phase forms a part of.

### Adaptation of tools and instruments

In 2018, the WHO introduced the BeSD framework and tools to assist policymakers, healthcare managers, and researchers in conducting a comprehensive analysis of the factors influencing vaccine acceptance and uptake [25]. Using these tools, programs can assess vaccine uptake, track trends over time, and systematically gather data to design, implement, and evaluate targeted interventions aimed at reducing hesitancy and improving vaccination rates.

The Vaccination Attitudes Examination (VAX) Scale is a tool designed to evaluate general attitudes toward vaccines. Its development focused on identifying anti-vaccination attitudes that are predictive of vaccination behavior [18]. Four key attitudes were identified: (1) mistrust of vaccine benefit, (2) worries over unforeseen future effects, (3) concerns about commercial profiteering, and (4) preference for natural immunity. These four factors cover a broad range of anti-vaccination sentiments, making the VAX scale highly effective in identifying individuals who are resistant to vaccination [26].

The BeSD framework and tools focus on measurable factors at the individual level, specifically related to vaccination. It is organized into four domains. The “thinking-and-feeling” domain examines individuals’ perceptions of the risks posed by diseases and their confidence in the vaccine, including their trust in its safety and benefits. The “social processes” domain explores the influence of social norms on vaccination, such as recommendations from healthcare providers. The “motivation” domain centers on individuals’ intention to get vaccinated, while the “practical issues” domain addresses their experiences with the vaccination process [25]. The full framework includes multiple questions for each domain, whereas a simplified version uses a single priority question per domain.

Although various factors impact vaccine decision-making, the BeSD framework is regarded as an effective tool for researchers, healthcare managers, and policymakers to better understand the motivations behind vaccination intentions and uptake. Its focus on measurable drivers allows it to guide decisions related to program implementation, monitoring, and evaluation [25].

The General Attitudes Toward Vaccine Survey questions was developed from the compendium of survey questions created by the WHO SAGE working group on vaccine hesitancy [17] and have been previously adapted to maternal vaccines [20,21].

The questionnaire assessing attitudes toward vaccination during pregnancy was developed from previously published studies and showed high validity [20–24]. Besides, the instrument has been used among pregnant women in Kenya [20,21].

The quantitative questionnaire covers participants’ demographics and obstetric history, the VAX scale [18], the BeSD of COVID-19 vaccination tools and the BeSD priority indicators for routine childhood vaccines, based on the WHO BeSD vaccination framework [25], the General Attitudes Toward Vaccine Survey questions [25], and the questionnaire assessing knowledge of influenza and attitudes toward influenza vaccination during pregnancy [20–24].

For the qualitative component, we developed an interview guide to assess participants’ understanding including attitudes and beliefs, and to test their ability to engage with the interview guide content using the following BeSD domains: “thinking and feeling”; “social processes”; “motivation”, and “pragmatic issues” [25].

At the start of the study, the English versions of the quantitative questionnaire and interview guide were used. Based on initial participant feedback regarding question clarity and comprehension, necessary adjustments were made to improve understanding. Once the questions were consistently understood, all study documents were translated into IsiXhosa and Afrikaans, two of the three official languages in the Western Cape, South Africa, and then back-translated into English to ensure accuracy and consistency [27]. The translated versions were pretested, and further refinements were made by native speakers. The adaptation process was supported by a bilingual researcher whose primary language is Afrikaans and a research assistant who is a native IsiXhosa speaker and fluent in English. Both have experience conducting research in health settings and assisted in refining terminology to ensure cultural relevance and accessibility.

Below is a detailed description of the changes made during the adaptation process:

- For the General Attitudes towards Maternal Vaccines, we applied minor changes to the questions for clarity as follows: “Vaccines given during pregnancy are important for my health,” “All recommended vaccines for pregnant women offered by the government program in my community are beneficial,” and “Recommended vaccines for pregnant women are effective.”
- For the VAX scale, a five-point Likert scale “Strongly Disagree”, “Disagree”, “Neutral”, “Agree”, “Strongly Agree” was used instead of a six-point Likert scale, “Strongly Disagree”, “Disagree”, “Somewhat disagree”, “Somewhat agree”, “Agree”, “Strongly Agree”.
- For the BeSD COVID-19 tool, the “Reasons for low ease of access,” “Service satisfaction,” and “Service quality” constructs were not included, as the focus of the study was on knowledge and attitudes. Most of the questions were adapted for use among pregnant women. For instance, “How concerned are you about getting COVID-19 during pregnancy?”, “Do you want to get a COVID-19 vaccine during pregnancy?”, and “How safe do you think a COVID-19 vaccine is for you as a pregnant woman?”. Minor changes to the multiple-choice options were applied to a few questions, such as “Have you ever been contacted about being due for a COVID-19 vaccine?” (answer options: Yes, before pregnancy; Yes, during this pregnancy; No), etc. We also added the following questions: “Has a health worker recommended you get a COVID-19 vaccine before pregnancy?” as participants might have received a health worker recommendation before pregnancy. We added a question about the reason for receiving the COVID-19 vaccine before pregnancy, a question on whether COVID-19 has been discussed with the participant during the current pregnancy, and a question on what will make the participant confident in accepting the COVID-19 vaccine during pregnancy.
- For the tool assessing knowledge of influenza and attitudes toward influenza vaccination during pregnancy, we added the term “Flu” alongside “Influenza,” as many people are more familiar with the term “flu” than “influenza”.
- We added “Yes or No” questions about trust in COVID-19, Influenza, and routine childhood vaccines to compare participants’ trust in the different vaccines.
- At the time of the study, the National Department of Health in South Africa was planning to switch from Tetanus-reduced diphtheria (Td), which had been administered during antenatal care, to Tetanus, reduced-strength diphtheria, and acellular pertussis (Tdap) starting in January 2024 [28]. Therefore, we provided the following information about pertussis: “Whooping cough is a highly contagious respiratory tract disease. Young babies are the most vulnerable group with the highest rates of complications and death. A safe and protective whooping cough vaccine is already available. It has proven to be safe and protective against whooping cough among young babies when pregnant women get vaccinated.” We asked participants if they would accept Tdap during pregnancy if it were offered to them.
- We also provided the participants with the following information about the Respiratory Syncytial Virus: “Respiratory Syncytial Virus (RSV) is a virus that can cause mild cold-like symptoms in adults but can cause more serious illness in young babies. It is the most common cause of lung infection (also known as bronchiolitis or pneumonia) in young babies. A vaccine for RSV to protect babies is being developed and has been trialed in adults and pregnant women.” We asked them whether they would accept receiving an RSV vaccine during pregnancy if an effective and safe vaccine against RSV becomes available to use among pregnant women.
- For the interview guide, the following BeSD constructs were explored for both COVID-19 and childhood vaccines: “What people feel and think”, “Motivation”, and “Social processes.”
- Questions about the following topics were added to the interview guide:
  - ◦ Questions about the participants’ knowledge and attitudes towards vaccines in general and vaccination during pregnancy.
  - ◦ Questions regarding COVID-19 vaccine decision-making before pregnancy to compare the participants’ attitudes before and during pregnancy.
  - ◦ We also discussed whether they would accept the Tdap vaccine after being informed about it.

### Data collection procedures

Quantitative data was collected through a self-administered structured questionnaire on the Research Electronic Data Capture (REDCap) web application [29,30]. The questionnaire had four sections: Section One relates to data on demographics and pregnancy; Section Two concerns participants’ attitudes towards vaccination in general and maternal vaccines; Section Three contains the WHO BeSD tool for COVID-19 vaccination, and the tool assessing knowledge of influenza and influenza vaccination during pregnancy; and Section Four contains the BeSD tool for childhood vaccination priority indicators. Participants completed the quantitative survey while they were waiting for their appointment with their healthcare providers in the waiting area.

We conducted semi-structured interviews using the adapted guide with the participants who agreed to participate in the qualitative component on the same day they completed the quantitative survey after they had seen their healthcare providers in a private room in the health facility that had been provided to the research staff.

### Data analysis

The analysis of both quantitative and qualitative data was approached through an integrated mixed-methods perspective, where the two parts complemented each other to address the study objectives. Quantitative data were analyzed using Stata software version 18.0 (Stata Corp., College Station, TX), with descriptive statistics employed to summarize demographic characteristics. Continuous variables were reported as means and standard deviations, while categorical variables were presented as proportions and percentages. For qualitative data, thematic analysis was applied, starting with verbatim transcription of interviews. Using NVivo software, codes were developed to cluster concepts within the data [31]. After identifying recurring codes, they were grouped into subcodes or categories, and themes were derived by connecting across participants and categories. This integrated approach allowed for a comprehensive understanding of the study’s findings, where the quantitative data provided statistical insights, and the qualitative analysis revealed deeper contextual and thematic insights, together offering a richer interpretation of the study objectives.

## Results

**Fig 1:**
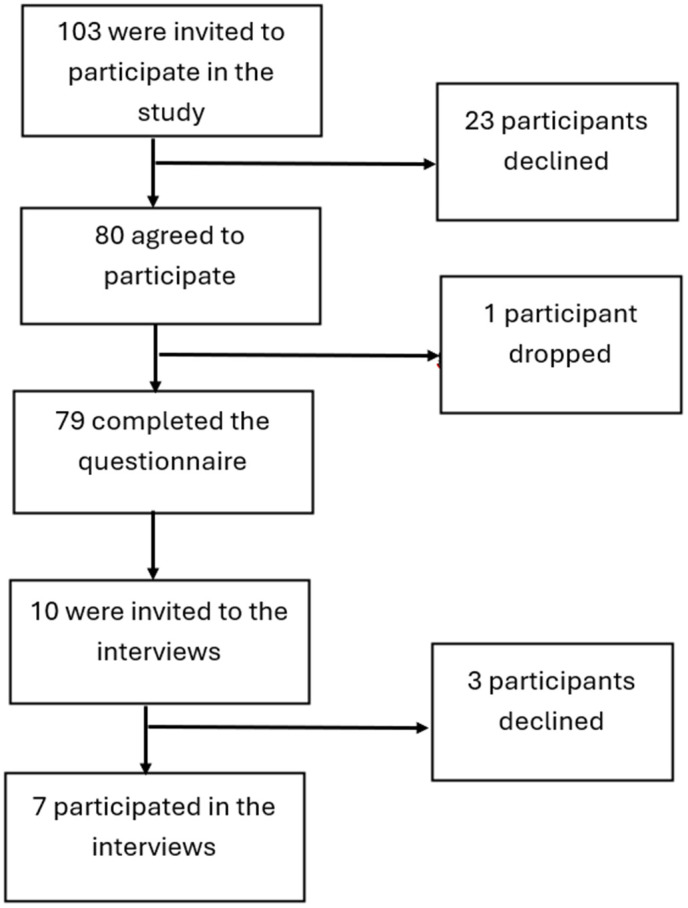
Enrollment flow chart.

A total of 103 participants were approached and invited to fill out the study questionnaire. Of these, 80 pregnant women agreed to participate and signed the consent form. Participants filled out the self-administered surveys in the waiting area while waiting for their appointment with their healthcare provider. During the study, only one participant dropped out. The response rate was 77% (79/103). Of the 79 participants who completed the questionnaire, 10 were further invited to participate in the qualitative component. Three participants declined, and seven agreed and participated in the interviews, signing the relevant consent form as shown in flow chart “Fig 1”. Interviews were conducted after the participants’ appointments, before they left the hospital, in a private room provided by the antenatal staff.

### Demographic characteristics of the study population

The mean age of the participants was 31 ± 6 years. Most pregnant women fell into the Black African (n = 47, 60%), and Colored (n = 29, 38%) categories. We determined maternal socio-economic status based on household income and the majority were in the low-income category (n = 76, 96%) and had a secondary level of education (n = 52, 66%).

The mean gestational age was 15 ± 8 weeks, 62% (n = 47) of the participants had high-risk pregnancies, and 14% (n = 11) were HIV positive as shown in “Table 1”.

**Table 1:** Demographic characteristics of the study population (n = 79).

Characteristic	n	%
**Age (years)**		
Mean (SD)	31 ± 6	–
**Gestational age (weeks)**		
Mean (SD)	15 ± 8	–
**Pregnancy risk status**		
High risk	47	62
Low risk	29	38
Race*		
Black African	47	59.4
Colored	29	36.7
White	1	1.3
Indian	1	1.3
Asian	1	1.3
**Educational level**		
Primary	2	2.5
Secondary	52	65.8
Post-secondary certificate	6	7.6
Tertiary	19	24.1
**Employment**		
Employed	55	70
Unemployed	24	30
**Household income**		
Low	76	96
Middle	3	4
**HIV status**		
HIV positive	11	13.9
HIV negative	68	86.1

SD = Standard deviation

*This refers to (i) self-identified race (ii) these categories stemmed from the Apartheid Era and are officially used to describe South Africans [32].

Filling out the questionnaire took an average of 17 minutes (range: 14–20 minutes). Interviews were conducted after the participants’ appointments, before they left the hospital, in a private room provided by the antenatal staff. The interviews lasted between 20–40 minutes.

### Participant feedback and usability of tools

The first twenty participants completed the English version of the questionnaire. A few of them did not understand the word “con” in the VAX scale, so we changed it to “scam.” The participants found the adapted survey questions clear overall and did not encounter any difficulties in answering the questions.

Participants found filling out the survey on the tablet easy and did not experience any difficulties, especially because all questions were either “Yes or No” or multiple-choice type questions. Besides, the participants found the information we provided on the Tdap and RSV vaccines helpful in answering the related questions.

The questionnaire was then translated into IsiXhosa and Afrikaans and back-translated into English. The remaining 60 participants were allowed to fill out the questionnaire in their preferred language. Only three participants chose the IsiXhosa version, and two chose the Afrikaans version.

Five participants did not complete the full questionnaire. To ensure completeness, we adjusted the survey settings on REDCap so that participants could not submit the survey if there were any unanswered questions.

Overall, participants found the survey easily understandable and filled it out without any difficulties. They provided minor comments on the translated versions, and we made the necessary changes.

The final version of the survey is provided in S1 File.

### Findings on general attitudes toward vaccination

Findings from the VAX scale indicated that 64.5% (n = 49) of the participants rely on vaccines to stop infectious diseases, and 62.7% (n = 47) feel protected after being vaccinated. Fifty-nine-point nine percent (n = 42) believe that most vaccines appear to be safe, though there may be problems that have not yet been discovered, and 46% (n = 35) believe that vaccines can cause unforeseen problems in children. Furthermore, 66% (n = 50) of the study participants reported that they worry about the unknown effects of vaccines in the future. Results of the VAX scale are presented in “Table 2”.

**Table 2:** Participants’ attitudes towards vaccination in general (n = 76).

No	Statement	Strongly Disagree	Disagree	Neutral	Agree	Strongly Agree
1	I feel safe after being vaccinated.	13 (17.1%)	5 (6.7%)	18 (23.7%)	20 (26.7%)	22 (29.0%)
2	I can rely on vaccines to stop serious infectious diseases.	8 (10.5%)	7 (9.2%)	12 (15.8%)	24 (31.6%)	25 (32.9%)
3	I feel protected after getting vaccinated.	7 (9.3%)	5 (6.7%)	16 (21.3%)	27 (36.0%)	20 (26.7%)
4	Although most vaccines appear to be safe, there may be problems that we haven’t yet discovered.	14 (18.4%)	3 (4.0%)	14 (18.4%)	19 (25.0%)	26 (34.2%)
5	Vaccines can cause unforeseen problems in children.	14 (18.4%)	8 (10.5%)	19 (25%)	24 (31.6%)	11 (14.5%)
6	I worry about the unknown effects of vaccines in the future.	7 (9.2%)	7 (9.2%)	12 (15.8%)	24 (31.6%)	26 (34.2%)
7	Vaccines make a lot of money for pharmaceutical companies but don’t do much for regular people.	15 (19.7%)	10 (13.2%)	24 (31.6%)	14 (18.4%)	13 (17.1%)
8	Authorities promote vaccination for financial gain, not for people’s health.	21 (27.6%)	12 (15.8%)	22 (29.0%)	11 (14.5%)	10 (13.2%)
9	Vaccination programs are a big scam.	23 (30.3%)	11 (14.5%)	21 (27.6%)	15 (19.7%)	6 (7.9%)
10	Natural immunity lasts longer than a vaccination.	11 (14.5%)	10 (13.2%)	29 (38.2%)	15 (19.7%)	11 (14.5%)
11	Natural exposure to viruses and germs gives the safest protection.	12 (15.8%)	11 (14.5%)	29 (38.2%)	14 (18.4%)	10 (13.2%)
12	Being exposed to diseases naturally is safer for the immune system than being exposed through vaccination.	13 (17.1%)	11 (11.8%)	31 (40.8%)	13 (17.1%)	10 (13.2%)

*Note:* n = 76 due to three missing responses.

Two themes emerged when we discussed vaccines in general with pregnant women. Theme one relates to knowledge and beliefs about vaccines. Most of the participants had heard about vaccines, but they expressed having basic knowledge and stated that they didn’t know much about vaccination.

*“I don’t know anything about them. I just know that they protect you from the viruses.” Participant #1.*

*“It helps to prevent you from getting sick…Or if you get it, you won’t get it so bad…That’s all I know.” Participant #6.*

Two participants shared their beliefs on the influence of religion on their views on vaccination. One participant mentioned that her religion doesn’t believe in vaccines, but her religious views or religious leaders do not influence her stance on vaccination, as she believes vaccines prevent her from getting ill. However, another participant said that her religious views do influence her beliefs about vaccines and that she considers what her religious leaders tell her and their advice.

*“In my religion, we don’t believe in vaccines and vitamins…I would take it still because I believe it prevents you from getting sick.” Participant #6.*

*“I would say yes, because like in some religions would say like, do not take the vaccine then we can always pray about it. Because when you’re sick, there’s only one person that can heal you. And it’s, it’s our lord. So, I would say I would consider, their opinion…I’ve got a pastor. So, he sends me to church. And he would always tell us like, you can go, but it’s your own decision to make. But you must also know that this is stuff that is here to test us. To see if you, if your faith is gonna be strong. So, you can have your vaccine. It’s not a problem, but you can also just pray. And ask God, to protect you and your family.” Participant #7.*

Theme two is about the vaccination decision-making process including opinion leaders or significant persons who influence the participants’ intent to vaccinate, adding to their information-seeking behavior. All participants stated that the doctor’s opinion influences their intent to vaccinate the most.

*“Because usually I listen to what the doctor says. Like I’m a hypertension person. If he tells me, you must take your tablets at 7 a.m. I usually do that on time. So, when it comes to my health it’s going to be better for me. My blood pressure will stay normal. Because I take it every day at the same time. So, I would say the doctor’s opinion on vaccination.” Participant #1.*

*“If it’s recommended to me by a doctor, then I will take it. But if it’s not by a doctor, then I will not”. Participant #3.*

In contrast, when participants were asked whether their friends’ or family’s views and opinions on vaccines influenced their attitudes and intent to vaccinate, participants expressed differing views.

*“…If they are against it that’s their problem. I will still go for it.” Participant #6.*

*“I would say my family’s opinion, yeah, would also make me not take it.” Participant #2.*

Participant #4 said that her decision would depend on the disease and the side effects of the vaccine: *“The reason is going to depend on what kind of disease is going to come and then I must know that vaccine is not going to make my life, maybe is not, I’m not going to be sick after doing this vaccine.”*

Besides, Participant #5 touched on her information-seeking behavior while deciding whether to get vaccinated: *“…I will look for information on Google and the clinic. Then I’ll ask the nurse or a doctor. I will show her what I researched about it, and I will ask her. If it’s like that or if it’s not like that. Then I would believe the doctor.”*

### Knowledge and attitudes toward maternal vaccination

Fifty-one percent (n = 38) of pregnant women agreed that all recommended vaccines for pregnant women offered by the government program in their community are beneficial, and 53% (n = 40) agreed that the recommended vaccines for pregnant women are effective. However, 61% (n = 46) were concerned about the serious side effects of vaccines. Participants’ general attitudes towards maternal vaccines are presented in “Table 3”. Moreover, forty-six percent (n = 34) of participants thought pregnant women should be vaccinated against influenza or flu and only thirty-nine percent (n = 29) believed that it is safe for pregnant women to receive the influenza vaccine during pregnancy.

**Table 3:** Participants attitudes towards vaccination during pregnancy (n = 75).

No	Statement	Agree	Neutral/No opinion	Disagree
1	Vaccines given in pregnancy are important for my health.	37 (49.3%)	26 (34.6%)	12 (16.1%)
2	All recommended vaccines for pregnant women offered by the government program in my community are beneficial.	38 (50.7%)	25 (33.3%)	12 (16.0%)
3	Recommended vaccines for pregnant women are effective.	40 (53.3%)	27 (36.0%)	8 (10.7%)
4	New vaccines carry more risks than older vaccines.	11 (14.7%)	44 (58.7%)	20 (26.7%)
5	Getting vaccines is a good way to protect myself from disease.	51 (68.0%)	15 (20.0%)	9 (12.0%)
6	I am concerned about serious adverse effects of vaccines.	46 (61.3%)	18 (24.0%)	11 (14.7%)
7	I do not need vaccines for diseases that are not common anymore.	22 (29.3%)	32 (42.7%)	21 (28.0%)

*Note:* n = 75 due to four missing responses.

Two themes emerged when discussing vaccination during pregnancy with participants. Theme three is about Knowledge of Vaccination During Pregnancy. All study participants stated that they were unaware of vaccinations recommended for pregnant women.

*“I don’t know anything.” Participant #2.*

*“I don’t know much about it; to be honest, I haven’t really seen people getting vaccinated during pregnancy. I think there might not be enough information about it. Because I’m six months in now. This is the first time I heard about getting vaccinated while pregnant. Because when you’re pregnant, they just tell you, don’t do this, don’t do that. You can’t take this medication; you can’t take that. So, you just stay away from everything. So, I don’t think there’s enough information about it.” Participant #5.*

Theme four is about the vaccine decision-making process during pregnancy. We discussed with pregnant women how they would decide whether to get vaccinated during pregnancy if recommended vaccines were available.

*“If it’s recommended by the doctor, then I would take it.” Participant #7*

Two participants said they would get the Tdap vaccine to protect their babies.

*“I will get it for the safety of my baby.” Participant #3*

*“I will take it because I want to protect the baby.” Participant #4*

However, Participant #6 stated that she would not accept any vaccines during pregnancy: *“I don’t believe in taking anything when pregnant.”*

In addition, we explored COVID-19 vaccine acceptance among pregnant women. We asked them how they would decide whether to get vaccinated against COVID-19 during pregnancy if it were recommended. Only two participants said they would take the vaccine if recommended by their doctor to protect their baby. Most participants were hesitant about getting vaccinated against COVID-19 during pregnancy because they wanted to be sure the vaccine was safe for both them and their babies.

*“I need to know that if I take this COVID vaccine right now, while I’m pregnant, it’s going to be safe, I’m not going to be sick, and it’s not going to cause a problem in my body.” Participant #4*

### Knowledge and attitudes toward routine childhood vaccines

Seventy-three-point four percent (n = 58) of the participants felt that childhood vaccines are very important for their child’s health, 59.5% (n = 47) wanted to vaccinate their child with all recommended vaccines in South Africa, and 31.7% (n = 25) wanted their child to get some of these vaccines.

Three themes emerged when discussing childhood vaccination with participants. Theme five relates to knowledge and the trusted sources of information regarding childhood vaccination. All participants had heard about routine childhood vaccines and immunization schedules for babies. They understood that childhood vaccines protect infants and young children from infectious diseases. Their most trusted sources of information on childhood vaccination were doctors, nurses, and healthcare providers at clinics.

*“I know that they should get it since the period of being small and that it helps them with their immune system and growing up.” Participant #1*

*“It’s important that the baby must get the vaccine. Because babies must not be getting the illness and get sick and sick.” Participant #4*

*“Trusted sources of information are my doctor and nurses, that’s all.” Participant #6*

Theme six is about the intention to vaccinate and the reasons. All participants stated that they planned to vaccinate their children and shared their motivations.

*“Because I want her to be protected.” Participant #2*

*“Because I want him must get healthy, not getting sick. Getting to cough, I don’t want to, I don’t want to lose him.” Participant #4*

*“Definitely. Because that is just my belief. Because I was raised that way, my mother made sure I was up to date. My siblings were up to date with our vaccines. I’ve already chosen for my son, my first baby, to have all these vaccines.” Participant #5*

Theme seven relates to the experience of vaccinating a child under five, one participant shared her experience of vaccinating her child, how she made the decision, and what she felt immediately after vaccinating her baby:

*“I have discussed it with no one. I just decided to do it...When my child was vaccinated, I felt relieved. Really relieved!”. Participant #5*

### COVID-19 vaccination knowledge and perceptions

COVID-19 vaccination BeSD priority indicators are presented in Table 4. Fifty-four-point four percent (n = 43) of the study participants felt that getting COVID-19 vaccine during pregnancy is “A little important” or “Not at all important.”

**Table 4:** COVID-19 vaccination BeSD priority indicators (n = 79).

Statement	Response options	n	%
- How important do you think getting a COVID-19 vaccine during pregnancy is for your health?			
Not at all important A little important Moderately important Very important		27	34.2
		16	20.3
		16	20.3
		20	25.3
- Do you want to get a COVID-19 vaccine during pregnancy?			
No, you do not want to Yes, you want to You are not sure No, I don’t want to because I am already vaccinated		54	68.4
		1	1.3
		3	11.4
		15	19.0
- Do you think most of your close family and friends want you to get a COVID-19 vaccine while you are pregnant?			
Yes No		18	22.8
		61	77.2
- Do you know where to go to get a COVID-19 vaccine for yourself?			
Yes No		59	74.7
		20	25.3
- How easy is it to pay for COVID-19 vaccination? When you think about the cost, please consider any payments to the clinic, the cost of getting there and the cost of taking time away from work?			
Not at all easy A little easy Moderately easy Very easy		9	11.4
		18	22.8
		13	16.5
		39	49.4

Sixty-eight-point four percent (n = 54) did not want to get the COVID-19 vaccine during the current pregnancy, and only one participant was willing to get vaccinated against COVID-19 during pregnancy.

Three themes emerged regarding COVID-19 disease and COVID-19 vaccines before getting pregnant. Theme eight focuses on participants’ perceived risk and concerns about contracting COVID-19. Most participants were worried about getting COVID-19, which they considered a severe disease.

*“I was scared of getting COVID because I heard that COVID is dangerous, it’s killing people. Yeah. I was very scared. “Participant #4.*

*“The very first time I was obviously very worried. Because I didn’t know much about it. I didn’t know what it was like. When we got COVID the first time, it was just off the road, and a shutdown happened. So, we didn’t, there wasn’t a lot of information out there. And I was very worried. Because my husband was very, very sick. We also had a son; my son was only a year or two at the time. And then uhm, the second time I got COVID, I had COVID and all these fevers. My son was very sick.” Participant #5.*

Participant #7 had different views and experiences with COVID-19. She was not worried, and when she and her family had COVID-19, it was mild, similar to the flu:

*“Everyone in the house had COVID. Yeah, so we didn’t even know it was COVID. And I went for a COVID test. When the test came back, I was fine you know. But it showed now we had COVID. But other than that, I wasn’t scared. I was just relaxed, I didn’t panic… Maybe it was self-esteem, because I said to myself, it was just the flu. So, I wasn’t scared. Nobody panicked. We were normal in the house. Everyone had it. So, it was normal for us. It was just like the flu.”*

Theme nine is on views and beliefs on COVID-19 vaccines. Participants shared different views on COVID-19 vaccines. For instance, one believed that COVID-19 vaccines are important because they prevent infection. Besides, another participant believed that COVID-19 vaccines are safe, and she has confidence in COVID-19 vaccines and the government.

*“Yeah, it’s important, because it makes us safe”. Participant #4.*

*“I think it’s very safe. Like I said, I don’t think people would put in the time and the effort. And all that money to then produce a product that is not good and not safe. I don’t think the government would do that to the people. And I don’t think it would be promoted on such a large scale.” Participant #5.*

Although Participant #7 was initially worried because COVID-19 vaccines were new, she later believed they were safe as she had not heard of anyone dying from the vaccine: *“With this new vaccine, I’m worried about safety... I think it is safe because I haven’t heard anyone died of it.”*

However, Participant #6 had negative views on COVID-19 vaccines, influenced by what she had heard from family and friends. *“I’ve heard a lot of stuff; I heard that it gives you the flu. I heard it from family, friends and like, they were complaining. Those who took the vaccine were complaining about, the pain they got in their arms. I don’t know if it is true because I never had it…I heard nothing positive.”*

Theme ten relates to the decision-making process for whether to get vaccinated or not against COVID-19 before the current pregnancy. The decision-making process regarding COVID-19 vaccination before the current pregnancy varied among participants. Participant #2 took the COVID-19 vaccine to protect herself from getting the disease: *“I just wanted to be protected from getting the Corona.”*

Participant #5 had a bad experience with COVID-19 and decided to get vaccinated after doing her research once the vaccine became available.

*“Because of how ill we were in the beginning. When we got COVID the first time. And then obviously, when the vaccine came out, we did our research. Is it safe? Is it good for us to take the vaccine? And it was then when I got COVID the second time around, it wasn’t that bad. I haven’t been sick even since it has been a few years now. Since I’ve had the vaccine.”*

However, Participant #3 chose not to get vaccinated due to concerns about side effects based on stories she had heard: *“I was very concerned. There were too many stories, like negative things people said on the internet, or friends of mine said very bad things, so I just decided not to do it.”*

## Discussion

The pilot testing of the quantitative self-administered survey and qualitative interviews was successfully conducted among pregnant women attending antenatal care in Cape Town. The survey methodology, the use of tablets, and logistics were effective. Pregnant women found the survey easy to complete, and the timing while waiting for their appointment was adequate. Conducting the interviews after their appointment with the healthcare provider was also convenient for them.

A key consideration is that data collection was conducted by a PhD student and a research assistant who were not part of the hospital staff. This made participants feel more comfortable and relaxed when sharing their opinions, particularly during qualitative interviews, as they were speaking with someone who was not a healthcare worker at the hospital they were attending, which may reduce the social desirability and interviewer bias.

This pilot study tested and adapted the WHO BeSD tools, the General Attitudes Toward Vaccine Survey questions developed by the WHO SAGE working group on vaccine hesitancy, a validated questionnaire on knowledge of influenza and attitudes toward influenza vaccination during pregnancy, and the VAX Scale, for use among pregnant women in Cape Town, South Africa. These tools have been used to explore maternal vaccine decision-making in countries like Kenya [20,21] Australia [33], and Poland [34] where pregnant women believed that a pregnant woman would be protected if vaccinated [20,21]. Mistrust of Vaccine Benefit, Concerns about Commercial Profiteering, and Preference for Natural Immunity constructs of the VAX scale were associated with vaccine uptake during pregnancy [34]. Additionally, pregnant women reported that they were concerned that the COVID-19 vaccine could cause harm to the unborn baby [33].

Despite the significant burden of vaccine-preventable diseases and critical gaps in vaccine delivery and uptake in LMICs [35], nuanced explorations of the knowledge, acceptance, and attitudes of pregnant women towards vaccines using these tools are inadequately undertaken or reported. This potentially undermines appropriate reforms to existing policy and practice. Our findings provide initial evidence of the tools’ usability and acceptability among pregnant women in South Africa while identifying areas for refinement to ensure their suitability for broader application in settings with similar contexts.

### Key findings and appraisal

The data collection tools were well-received by participants, who reported ease of use and understanding, particularly when using tablets for the quantitative survey. This is consistent with findings from other studies that demonstrate the feasibility of self-administered, tablet-based surveys in low-resource settings for capturing maternal health data efficiently [36–38]. Importantly, the adapted tools were sensitive enough to capture nuanced attitudes toward maternal and routine childhood vaccination, as well as participants’ perceptions of COVID-19 vaccines.

The qualitative data corroborated the survey findings. For instance, 61% of participants expressed concern about vaccine side effects (Table 3), which is echoed in Participant #4’s statement: “I was concerned about serious adverse effects…” Furthermore, 51% of pregnant women agreed that all maternal vaccines offered by the government are beneficial, and 53% believed these vaccines are effective (Table 3). This perception is reflected in Participant #3’s expression: “I will get it for the safety of my baby.” In addition, 73% of study participants stated that routine childhood vaccines are important for their child’s health, a view that is clearly illustrated by Participant #1’s opinion: “I know that they should get it since the period of being small and that it helps them with their immune system and growing up.”

Besides, quantitative results revealed moderate levels of vaccine confidence, with notable concerns about vaccine safety during pregnancy. Qualitative data underscored the critical role of healthcare providers as trusted sources of information, corroborating existing literature that identifies healthcare provider recommendations as pivotal in maternal vaccine decision-making [16,35]. However, knowledge gaps regarding specific vaccines recommended during pregnancy were evident, emphasizing the need for targeted educational interventions. For instance, pregnant women participating in the interviews indicated that they were not aware of any vaccines recommended during pregnancy. However, the National Integrated Maternal and Perinatal Care Guidelines for South Africa provide comprehensive guidelines on vaccines recommended for pregnant women [39]. These vaccines including Td, Influenza, and COVID-19 are administered to all pregnant women at no cost at public health facilities. Very recently, the National Department of Health switched from Td to Tdap to enhance protection against pertussis [28]. Further additions to existing guidelines include recommendations on COVID-19 vaccines [40,41]. Unfortunately, a low level of awareness among pregnant women regarding these vaccine recommendations negatively impacts demand generation and uptake. Further to this, gaps in service delivery, such as stockouts of COVID-19 or flu vaccines create barriers to acceptance and uptake [42,43].

In addition, our findings demonstrate the utility of the VAX Scale and BeSD tools in eliciting pregnant women’s perceptions of vaccine safety and effectiveness, but some adaptations were required to enhance their cultural and contextual relevance. For example, minor linguistic changes improved comprehension of key terms like “con,” which was revised to “scam”. Such modifications highlight the importance of pretesting tools to ensure clarity and accuracy in diverse settings.

Lastly, previous studies that employed the same tools used in our research have demonstrated their reliability, often with only minor modifications. For example, a study conducted in Australia aimed to identify factors influencing the adult population’s intention to receive the influenza vaccine using a slightly adapted version of the BeSD tool. The psychometric evaluation of the modified survey revealed strong validity within the Australian context [44]. Similarly, research carried out in South Africa to explore predictors of COVID-19 vaccine hesitancy in local communities found the BeSD tool effective for use in the Global South [45]. This was confirmed after piloting the tool in the local context and applying minor adjustments [45]. Furthermore, a study in Kenya investigating attitudes toward maternal vaccines utilized the same instruments as our study to assess general attitudes toward maternal vaccination, knowledge of influenza, and perceptions of influenza vaccination during pregnancy [46]. The questionnaire was found to be reliable following pilot testing and subsequent adjustments based on participant feedback [46]. In addition, the VAX scale has been validated in South Africa by Padmanabhanunni et al., who reported satisfactory reliability and provided evidence for its construct, convergent, and predictive validity [47]. They concluded that the robust psychometric properties of the VAX scale in a low- to middle-income country setting could significantly contribute to advancing research and immunization policy, enabling more targeted interventions to improve vaccine uptake [47].

### Strengths and limitations of this pilot study

This study represents the first attempt to adapt and test these globally recognized tools for assessing vaccine attitudes among pregnant women in South Africa, filling a critical gap in the literature. The integration of quantitative and qualitative methodologies using a mixed-methods design allowed for a comprehensive evaluation of the tools’ performance and enriched the understanding of maternal vaccine confidence in the local context.

We also share practical insights with participant feedback on the tools’ usability and logistics offering valuable guidance for future research, such as leveraging waiting times at healthcare facilities for data collection. Further to this, while interviewing a sub-population of the survey participants provided crucial findings, we observed that prior participation in the survey may have influenced interviewees’ responses. We therefore propose interviewing a separate group of pregnant women with no prior knowledge of the survey questions to enhance insights on these focal areas.

As a pilot study, the sample size was limited, which may affect the generalizability of the findings. However, the primary goal was to refine the tools rather than draw definitive conclusions. In addition, while the tools were translated into multiple languages, most participants completed the survey in English, limiting insights into the effectiveness of the IsiXhosa and Afrikaans versions. Besides, this study was conducted in public antenatal clinics, and the findings may not reflect the experiences of pregnant women accessing private healthcare. Another limitation that may also affect the generalizability of the findings is that the study was conducted exclusively in the Cape Metro region of Cape Town. Consequently, the results may not be fully generalizable to rural areas, other provinces in South Africa, or to other LMICs. Furthermore, while the tools used in this study were applied within the South African context, they may require pilot testing and adaptation to ensure their relevance and effectiveness in other LMIC settings, particularly those with different healthcare infrastructures, cultural norms, or patient populations. Researchers may build on the findings of this pilot study and the larger cohort study to inform the design and contextualization of similar studies, especially within LMIC contexts.

Moreover, based on qualitative feedback from participants, the time required to complete the survey was generally perceived as reasonable and not overly long, especially considering that participants were recruited while often waiting several hours to be seen by healthcare providers. However, it is still possible that some individuals may have experienced respondent fatigue while completing the survey.

Additionally, the sampling of only secondary and tertiary hospitals may limit the generalizability of the findings to other healthcare settings. Another limitation is the use of phone-tablets for self-administered questionnaires, which may have been influenced by participants’ education levels. Moreover, future research should consider employing multivariate analysis or cohort study designs to strengthen the evidence base.

### Implications for future research

The results suggest that the WHO BeSD Framework and tools in addition to the VAX Scale, and the adapted General Vaccine Attitude Survey questionnaire that has been developed from the compendium of survey questions by the SAGE Working Group on Vaccine Hesitancy with minor adaptations, are fit for purpose in the South African context. Future studies should apply these tools in larger and more diverse populations to validate their utility and explore the broader determinants of vaccine acceptance. Additionally, efforts should be made to address the knowledge gaps identified in this pilot, particularly through healthcare provider-driven educational interventions.

## Conclusion

This pilot study demonstrates the feasibility and acceptability of using the WHO BeSD tools and framework, the General Attitudes Toward Vaccine Survey questions developed by the WHO SAGE working group on vaccine hesitancy, and the VAX Scale to assess vaccine confidence among pregnant women in South Africa. These findings pave the way for their use in larger-scale studies and contribute to the global understanding of maternal vaccine attitudes. Addressing maternal knowledge gaps and leveraging healthcare provider influence will be crucial in designing effective interventions to promote vaccine confidence and uptake in this and similar settings.

## Supporting information

S1 File The adapted study questionnaire. (DOCX)

S2 File Pilot dataset. (XLSX)

S3 File Themes and codes from the interviews with the corresponding quotes. (DOCX)
