# Supplementary material for: Adapting the WHO Behavioural and Social Drivers of Vaccination (BeSD) tools and Vaccination Attitudes Examination (VAX) scale for pregnant women in South Africa: Insights from a mixed-methods pilot study
Source: PLoS One. 2025 Nov 5;20(11):e0334854. doi: 10.1371/journal.pone.0334854 (PMC12588488; doi:10.1371/journal.pone.0334854)
Supplement: S1 File — (DOCX) [file pone.0334854.s001.docx]

Supporting information S1: the adapted study questionnaire

Please complete the survey below. Thank you!

Study ID

Name and Surname

Participation date

**DEMOGRAPHICS**

- **What is your education level?** None, Primary, Secondary, Post-secondary (post-matric certificate), Tertiary (University, College ...)
- **What are you currently doing?** Employed, Unemployed
- **Approximately what is the total monthly income for your family? The total income for your household including salaries, unemployment, child grants, etc..:** R 000 - 4.500, R 4.500 -12.700,

R 12.700 -30.300, R 30.300 -52.600, R 52.600 - 72.000, R 72.000 -110.800, R 110.800+.

- **What is your marital status?** Single/ Married /living with partner/ Divorced / Widowed
- **How many weeks are you pregnant?** (gestational age by weeks)
- **What is your race?** Asian, Indian, African, Coloured, White
- **Pregnancy risk status:**  High risk, Low risk
- **Do you have HIV?** Yes, No
- **COVID-19 BeSD tool including additional questions and routine childhood vaccination BeSD priority indicators**

| Statement | Response options |
| --- | --- |
| - To your knowledge, have you ever had COVID-19? | Yes  No |
| - Was it: | Confirmed by a test  Not confirmed by a test |
| - Did you receive a COVID-19 vaccine before pregnancy? | No  Yes you received one dose  Yes you received two doses  Yes you received three or more doses.  NOT SURE |
| - In the past, did you have any unpleasant vaccine side effects with other vaccines? | Yes  No |
| - What was your main reason for receiving/ or not receiving the COVID-19 vaccine before pregnancy? Select all that apply (you can select many answers) | - My understanding of the disease and vaccination  - Information from my doctor/hospital  - Information from social media  - Information from news  - Information from family/friends  - Information from a religious leader  - Information from a political leader  - It was compulsory at work to get vaccinated |
| - Has the COVID-19 Vaccine been discussed with you by any health worker during this pregnancy? Select all that apply (you can select many answers) | -Midwife/Nurse  - Doctor/ Obstetrician  - Unsure if it has been discussed with me  - COVID-19 vaccination has not yet been discussed with me |
| - Has a health worker recommended you get a COVID-19 vaccine before pregnancy? | Yes  No |
| - Have you ever been contacted about being due for a COVID-19 vaccine? | Yes before pregnancy  Yes during this pregnancy  No |
| -How concerned are you about getting COVID-19 during pregnancy? | Not at all concerned  A little concerned  Moderately concerned  Very concerned |
| - How important do you think getting a COVID-19 vaccine during pregnancy is for your health? |  |
|  | Not at all important |
|  | A little important |
|  | Moderately important |
|  | Very important |
| - Do you want to get a COVID-19 vaccine during pregnancy? |  |
|  | No, you do not want to |
|  | Yes you want to |
|  | You are not sure |
|  | No, I don't want to because I am already vaccinated |
| - How safe do you think a COVID-19 vaccine is for you as a pregnant woman? | Not at all safe  A little safe |
|  |  |
|  |  |
|  |  |
|  |  |
| - How much do you trust the health workers who would give you a COVID-19 vaccine? | Not at all  A little  Moderately  Very much |
| - Do you think most of your close family and friends want you to get a COVID-19 vaccine while you are pregnant? | Yes  No |
| - Do you think your religious leaders want you to get a COVID-19 vaccine while you are pregnant? | Yes  No |
| - Do you think other community leaders want you to get a COVID-19 vaccine while you are pregnant? | Yes  No |
| - Has a health worker recommended you get a COVID-19 vaccine during this pregnancy? | Yes  No |
| - Do you think most of the people you work with got a COVID-19 vaccine? | No  Yes  NOT CURRENTLY WORKING |
| - If it was for you to get a COVID-19 vaccine, would you need permission to go and get it? | Yes, I need a permission to go and get COVID-19 vaccine.  No, I don't need permission I can decide on my own. |
|  |  |
| - Do you know where to go to get a COVID-19 vaccine for yourself? |  |
|  | Yes |
|  | No |
| - Is a COVID-19 vaccine available for you to get at your place of work? | No  Yes  NOT CURRENTLY WORKING |
| - Is a COVD-19 vaccine available for you to get at your health facility where to have an antenatal care? | Yes  No  I don’t know |
|  |  |
| - How easy is it to pay for COVID-19 vaccination? When you think about the cost, please consider any payments to the clinic, the cost of getting there, and the cost of taking time away from work. |  |
|  | Not at all easy |
|  | A little easy |
|  | Moderately easy |
|  | Very easy |
| - How important do you think routine childhood vaccines (immunization) are for your child's health? |  |
|  | Not at all important |
|  | A little important |
|  | Moderately important |
|  | Very important |
|  |  |
| - Do you think most of your close family and friends want you to get your child vaccinated? |  |
|  | Yes |
|  | No |
|  |  |
| - South Africa has an immunization schedule of recommended vaccines for children. Do you want your child to get none of these vaccines (immunization), some of these vaccines (immunization), or all of these vaccines (immunization)? |  |
|  | None |
|  | Some |
|  | All |
| - Do you know where to go to get your child vaccinated? |  |
|  | Yes |
|  | No |
| - How easy is it to pay for your child vaccination? When you think about the cost, please consider any payments to the clinic, the cost of getting there, plus the cost of taking time away from work? (Think of any related cost even if the vaccines will be given for free at the clinic that you will go to get your child vaccinated) |  |
|  | Not at all easy |
|  | A little easy |
|  | Moderately easy |
|  | Very easy |
|  |  |

- **The General Attitudes towards Vaccination, participants were asked to choose one answer among the following options: “Strongly Disagree”, “Disagree”, “Neutral “, “Agree”, or “Strongly Agree”:**

| No | Statement |
| --- | --- |
| 1 | I feel safe after being vaccinated. |
| 2 | I can rely on vaccines to stop serious infectious diseases. |
| 3 | I feel protected after getting vaccinated. |
| 4 | Although most vaccines appear to be safe, there may be problems that we haven't yet discovered. |
| 5 | Vaccines can cause unforeseen problems in children. |
| 6 | I worry about the unknown effects of vaccines in the future. |
| 7 | Vaccines make a lot of money for pharmaceutical companies but don't do much for regular people. |
| 8 | Authorities promote vaccination for financial gain, not for people's health. |
| 9 | Vaccination programs are a big scam. |
| 10 | Natural immunity lasts longer than a vaccination. |
| 11 | Natural exposure to viruses and germs gives the safest protection. |
| 12 | Being exposed to diseases naturally is safer for the immune system than being exposed through vaccination. |

- **The VAX scale participants were asked to choose one answer among the following options: “Agree”, “Neutral/ No opinion”, or “Disagree”:**

| No | Statement |
| --- | --- |
| 1 | Vaccines given in pregnancy are important for my health. |
| 2 | All recommended vaccines for pregnant women offered by the government program in my community are beneficial. |
| 3 | Recommended vaccines for pregnant women are effective. |
| 4 | New vaccines carry more risks than older vaccines. |
| 5 | Getting vaccines is a good way to protect myself from disease. |
| 6 | I am concerned about serious adverse effects of vaccines. |
| 7 | I do not need vaccines for diseases that are not common anymore. |

- **Knowledge of influenza and attitudes toward influenza vaccination questionnaire, participants were asked to choose one answer among the following options: “Yes”, “No”, or “Not sure”:**

| No | Statement |
| --- | --- |
| 1 | Have you ever heard about influenza or flu? |
| 2 | Do you think that a pregnant woman should be vaccinated against influenza (flu)? |
| 3 | If you were given the option to get a vaccine against influenza (flu), would you accept vaccination? |
| 4 | Is it likely for a pregnant woman who has not been vaccinated against influenza (flu) to get the disease? |
| 5 | Is a pregnant woman protected if she is vaccinated against influenza (flu)? |
| 6 | Do you think it is safe for a pregnant woman to receive influenza (flu) vaccine? |
| 7 | Would a baby after being born be protected against influenza, if his/her mother received an influenza (flu) vaccine during pregnancy? |

- **Questions about trust in vaccines:**
- Do you trust COVID-19 vaccines?
- Do you trust the influenza (flu) vaccine?
- Do you trust routine childhood vaccines? (Such as BCG, MMR, OPV HepB etc...)
- **Questions about acceptance of Tdap and RSV:**
- "*Whooping cough is a highly contagious respiratory tract disease. Young babies are the most vulnerable group with the highest rates of complications and death. A safe and protective whooping cough vaccine is already available. It has proven to be safe and protective against whooping cough among young babies when pregnant women get vaccinated*".

Would you like to receive a vaccine against whooping cough during pregnancy if it is available for you?

- *“Respiratory Syncytial Virus (RSV) is a virus that can cause mild cold-like symptoms in adults but can cause more serious illness in young babies. It is the most common cause of a lung infection (also known as bronchiolitis or pneumonia) in young babies. A vaccine for RSV to protect babies is being developed and has been trialled in adults and pregnant women".*

Would you like to receive a vaccine that protects against RSV if an effective and safe vaccine against RSV becomes available to use among pregnant women?"
