# Supplementary material for: Adapting the WHO Behavioural and Social Drivers of Vaccination (BeSD) tools and Vaccination Attitudes Examination (VAX) scale for pregnant women in South Africa: Insights from a mixed-methods pilot study
Source: PLoS One. 2025 Nov 5;20(11):e0334854. doi: 10.1371/journal.pone.0334854 (PMC12588488; doi:10.1371/journal.pone.0334854)
Supplement: S3 File — (DOCX) [file pone.0334854.s003.docx]

**General Attitudes Toward Vaccination**

**knowledge and beliefs about vaccines**

*“I don't know anything about them. I just know that they protect you from the viruses.” Participant #1.*

*“ It helps to prevent you from getting sick….Or if you get it, you won't get it so bad…That's all I know.” Participant #6.*

*“In my religion, we don't believe in vaccines and vitamins…I would take it still because I believe it prevents you from getting sick.” Participant #6.*

*“I would say yes, because like in some religions would say like, do not take the vaccine then we can always pray about it. Because when you're sick, there's only one person that can heal you. And it's, it's our lord. So, I would say I would consider, their opinion…I've got a pastor. So, he sends me to church. And he would always tell us like, you can go, but it's your own decision to make. But you must also know that this is stuff that is here to test us. To see if you, if your faith is gonna be strong. So, you can have your vaccine. It's not a problem, but you can also just pray. And ask God, to protect you and your family.” Participant #7.*

**Vaccination decision-making process**

*“Because usually I listen to what the doctor says. Like I'm a hypertension person. If he tells me, you must take your tablets at 7 a.m. I usually do that on time. So, when it comes to my health it's going to be better for me. My blood pressure will stay normal. Because I take it every day at the same time. So, I would say the doctor's opinion on vaccination.” Participant #1.*

*“If it's recommended to me by a doctor, then I will take it. But if it's not by a doctor, then I will not”. Participant #3.*

*“…If they are against it that's their problem. I will still go for it.” Participant #6.*

*“I would say my family's opinion, yeah, would also make me not take it.” Participant #2.*

*“ The reason is going to depend on what kind of disease is going to come and then I must know that vaccine is not going to make my life, maybe is not, I'm not going to be sick after doing this vaccine.” Participant #4.*

*“…I will look for information on Google and the clinic. Then I'll ask the nurse or a doctor. I will show her what I researched about it, and I will ask her. If it's like that or if it's not like that. Then I would believe the doctor.” Participant #5.*

**Knowledge and Attitudes Toward Maternal Vaccination**

**Knowledge of Vaccination During Pregnancy**

*“I don't know anything.” Participant #2.*

*“I don't know much about it; to be honest, I haven't really seen people getting vaccinated during pregnancy. I think there might not be enough information about it. Because I'm six months in now. This is the first time I heard about getting vaccinated while pregnant. Because when you're pregnant, they just tell you, don't do this, don't do that. You can't take this medication; you can't take that. So, you just stay away from everything. So, I don't think there's enough information about it.” Participant #5.*

**Vaccine decision-making process during pregnancy**

*“If it's recommended by the doctor, then I would take it.” Participant #7*

*“I will get it for the safety of my baby. ” Participant #3*

*“I will take it because I want to protect the baby.” Participant #4*

*“I don't believe in taking anything when pregnant.” Participant #6*

*“I need to know that if I take this COVID vaccine right now, while I'm pregnant, it's going to be safe, I'm not going to be sick, and it's not going to cause a problem in my body.” Participant #4*

**Knowledge and Attitudes Toward Routine Childhood Vaccines**

**knowledge and the trusted sources of information regarding childhood vaccination**

*“I know that they should get it since the period of being small and that it helps them with their immune system and growing up.” Participant #1*

*“It's important that the baby must get the vaccine. Because babies must not be getting the illness and get sick and sick.” Participant #4*

*“Trusted sources of information are my doctor and nurses, that's all.” Participant #6*

**Intention to vaccinate and the reasons**

*“Because I want her to be protected.” Participant #2*

*“Because I want him must get healthy, not getting sick. Getting to cough, I don't want to, I don't want to lose him.” Participant #4*

*“Definitely. Because that is just my belief. Because I was raised that way, my mother made sure I was up to date. My siblings were up to date with our vaccines. I've already chosen for my son, my first baby, to have all these vaccines.” Participant #5*

**Experience of vaccinating a child under five**

*“I have discussed it with no one. I just decided to do it...When my child was vaccinated, I felt relieved. Really relieved!”. Participant #5*

**COVID-19 Vaccination Knowledge and Perceptions**

**Perceived risk and concerns about contracting COVID-19**

*“I was scared of getting COVID because I heard that COVID is dangerous, it's killing people. Yeah. I was very scared. “Participant #4.*

*“The very first time I was obviously very worried. Because I didn't know much about it. I didn't know what it was like. When we got COVID the first time, it was just off the road, and a shutdown happened. So, we didn't, there wasn't a lot of information out there. And I was very worried. Because my husband was very, very sick. We also had a son; my son was only a year or two at the time. And then uhm, the second time I got COVID, I had COVID and all these fevers. My son was very sick.” Participant #5.*

*”Everyone in the house had COVID. Yeah, so we didn't even know it was COVID. And I went for a COVID test. When the test came back, I was fine you know. But it showed now we had COVID. But other than that, I wasn't scared. I was just relaxed, I didn't panic… Maybe it was self-esteem, because I said to myself, it was just the flu. So, I wasn't scared. Nobody panicked. We were normal in the house. Everyone had it. So, it was normal for us. It was just like the flu.” Participant #7*

**Views and beliefs on COVID-19 vaccines**

*“Yeah, it's important, because it makes us safe”. Participant #4.*

*“I think it's very safe. Like I said, I don't think people would put in the time and the effort. And all that money to then produce a product that is not good and not safe. I don't think the government would do that to the people. And I don't think it would be promoted on such a large scale. If It's a good potential to be harmful.” Participant #5.*

*“With this new vaccine, I'm worried about safety... I think it is safe because I haven't heard anyone died of it.”* *Participant #7*

*“I've heard a lot of stuff; I heard that it gives you the flu. I heard it from family, friends and like, they were complaining. Those who took the vaccine were complaining about, the pain they got in their arms. I don't know if it is true because I never had it…I heard nothing positive.” Participant #6*

**Decision-making process for whether to get vaccinated or not against COVID-19 before the current pregnancy**

*“I just wanted to be protected from getting the Corona.” Participant #2*

*“Because of how ill we were in the beginning. When we got COVID the first time. And then obviously, when the vaccine came out, we did our research. Is it safe? Is it good for us to take the vaccine? And it was then when I got COVID the second time around, it wasn't that bad. I haven't been sick even since it has been a few years now. Since I’ve had the vaccine.” Participant #5*

*“I was very concerned. There were too many stories, like negative things people said on the internet, or friends of mine said very bad things, so I just decided not to do it.” Participant #3*
